# The Manifestations of Hysteria

**Published:** 1904-05-21

**Authors:** 


### THE MANIFESTATIONS OF HYSTERIA.

The concomitants of hysteria are discussed in a clinical supplement to the *London Hospital Gazette*. The symptoms, though they are legion, fall for the most part into sufficiently definite groups. These are dealt with seriatim.

Speech may be affected in a variety of ways: there is the well-known hysterical aphonia, in which the patient speaks in a whisper only, but when told to cough does so with a normal sound. If the vocal cords are examined they will be found to be widely separated in phonation, but they move perfectly well with respiration. Other varieties of speech affection are the hysterical stutter and hysterical mutism.

Convulsive attacks are usually classified as minor and major. In the former consciousness is retained, the tongue is not bitten, and there is no involuntary evacuation of urine. Although violent struggling may be present the movements are of the voluntary type and the clonic movements do not resemble those of true epilepsy. A major hysterical fit, the so-called hystero-epilepsy, is rare. In a well-marked example the patient is first thrown into a violent tonic contraction, in which the back may be arched to such an extent that she rests only on her face and her heels. This is followed by a period of violent clonic movements, which may end in an emotional outbreak. Sometimes the patient falls into attitudes expressive of some emotional state, and may lie quiescent, with her arms outstretched in what is known as the crucifixion attitude, or may kneel with the hands clasped as if in prayer; at other times the emotional outburst is noisy in character, and the patient sings or shouts. Such attacks may last for hours, but they can usually be cut short if the patient be made to vomit by the administration of apomorphine.

The motor manifestations of hysteria fall under the headings of tremor, spasm, and paralysis. The tremor may be a clonic movement, each phase of which is strictly repeated, and is in this case usually limited to one limb. Such movements increase while the subject is under observation and cease during sleep. But the tremor may be a true intention tremor, only appearing on voluntary movement. Such a tremor somewhat resembles that which characterises disseminated sclerosis, but differs from it in the fact that the patient seems unable to reach the spot aimed at. Spasm includes the majority of motor phenomena in hysteria, and almost any part of the body may be affected. When there is paralysis, there is never any wasting of a single group of muscles, and the muscles always respond to the Faradic current. Sometimes the patient is unable to walk or to stand, although he is capable of making all the necessary movements powerfully when lying in bed.

Pain in hysteria may arise in many different ways. Hysteria diminishes the resistance to pain impulses, and therefore the organic disturbance of some organ may produce widespread or intense pain and tenderness out of all proportion to the original cause of the disturbance. An aching tooth may be accompanied by tenderness over the greater part of one half of the body, or over the whole head and neck. A second variety of pain is more truly an outcome of an hysterical state, and is probably mental in origin. The patient may complain of pain in the knee, and the whole knee will be found to be tender to touch and to pressure. Or the patient may complain of pain over various parts of the body, and these parts will be found extremely tender even to the lightest touch. When anæsthesia is present the characteristic form is where touch, pain, heat and cold are lost over one half of the body or over one segment of the affected limb. This geometrical arrangement is characteristic of hysteria. The loss of sensation is bounded by a line traced round the limb, usually in the neighbourhood of a joint. Coterminous loss to all forms of sensation bounded by a line drawn across the limb in the neighbourhood of a joint is diagnostic of hysteria.

Any of the organs of special sense may be involved, though hearing is but rarely affected. Smell and taste are frequently abolished, while limitation of the field of vision is one of the most valuable indications of hysteria. It occurs in a large number of cases where its existence would be unsuspected from the other symptoms. The form assumed by the loss differs fundamentally from that produced by organic or functional disease. For in the normal retina an area is present over which colours are appreciated. Outside this area colours appear as a lighter or darker grey. Now in organic disease of the eye, however much the field of vision be diminished, this colour-blind zone remains. Hysterical limitation of the field of vision differs from this condition in that, if a coloured patch is seen at all, it is seen in its true colour.

The knee-jerks may be exaggerated, but are never absent on reinforcement. Ankle clonus is never present. If the plantar reflex can be elicited it is always normal in character. The pharyngeal reflex is frequently abolished, so that the finger can be thrust down to feel the upper opening of the larynx without giving the patient any obvious discomfort. The corneal reflex is rarely abolished. Anorexia and vomiting are frequent symptoms, and retention of urine is one of the commonest of concomitants of hysteria.

